# Supplementary material for: Cd2+ Sorption Alterations in Ultisol Soils Triggered by Different Engineered Nanoparticles and Incubation Times
Source: Nanomaterials (Basel). 2023 Dec 11;13(24):3115. doi: 10.3390/nano13243115 (PMC10745855; doi:10.3390/nano13243115)
Supplement: Supplementary file 1 [file nanomaterials-13-03115-s001.zip › nanomaterials-2727977-supplementary.pdf]

Supporting Information

Cd^{2+} Sorption Alterations in Ultisol Soils Triggered by Different Engineered Nanoparticles and Incubation Times

Karen Manquián-Cerda [1,*], Raúl Calderón [2], Mauricio Molina-Roco [3], Tamara Maldonado [4] and Nicolás Arancibia-Miranda [1,*]

[1] Facultad de Química y Biología, Universidad de Santiago de Chile, Av. B. O'Higgins, 3363, Santiago 9170124, Chile
[2] Centro de Investigación en Recursos Naturales y Sustentabilidad, Universidad Bernardo O'Higgins, Fabrica 1990, Segundo Piso, Santiago 8370993, Chile; raul.calderon@ubo.cl
[3] Departamento de Acuicultura y Recursos Agroalimentarios, Campus Osorno-Chuyaca, Universidad de los Lagos, Osorno 5290000, Chile; mauricio.molina1@ulagos.cl
[4] Instituto de Química, Facultad de Ciencias, Pontificia Universidad Católica de Valparaíso, Av. Universidad 330, Placilla, Valparaíso 2373223, Chile; tamara.maldonado@pucv.cl
* Correspondence: karen.manquian@usach.cl (K.M.-C.); nicolas.arancibia@usach.cl (N.A.-M.)

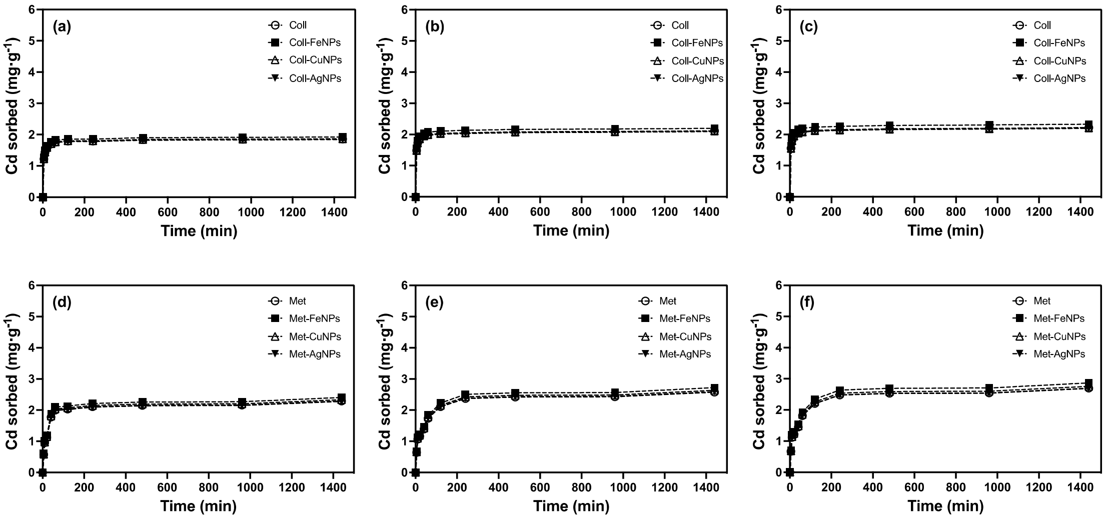

Figure S1. Effect of nanoparticle type and aging time on Cd sorption kinetics in Ultisols soils at a dose of 20 mg ENPs·kg^{-1}. Figure S1a and S1d Collipulli and Metrenco 1 month, Figure S1b and S1e Collipulli and Metrenco 3 months, and S1c and S1f Collipulli and Metrenco 6 months.

Table S1: Kinetic parameters predicted from the pseudo-second order for control soils and soils treated with 20 mg ENPs·kg^{-1}.

Collipulli

	1 Month				3 Month				6 Month			
**Treatment**	**Control**	**AgNPs**	**CuNPs**	**FeNPs**	**Control**	**AgNPs**	**CuNPs**	**FeNPs**	**Control**	**AgNPs**	**CuNPs**	**FeNPs**
q_{exp} ***(mg·g^{-1})***	1,73 ± 0,02	1,79 ± 0,01	1,82 ± 0,01	1,85 ± 0,02	1,81 ± 0,01	1,89 ± 0,01	1,91 ± 0,03	1,93± 0,02	1,85 ± 0,01	1,90 ± 0,01	1,93 ± 0,01	1,99 ± 0,01
q_{exp} ***(%)***	43,3 ± 2,1	44, 8 ± 1,9	45,2 ± 3,0	46,3 ± 2,1	45,1 ± 2,3	47,3 ± 3,1	47,8 ± 3,1	48,3 ± 1,3	46,3 ± 2,5	47,5 ± 3,3	48,3 ± 2,2	49,7 ± 1,1
**Parameters**	**1 Month**				**3 Month**				**6 Month**			
q_e ***(mg·g-1)***	1,84 ± 0,01	1,82 ± 0,01	1,84 ± 0,01	1,90 ± 0,01	1.92 ± 0,01	1,96 ± 0,02	2,07 ± 0,01	2,16 ± 0,01	2,18 ± 0,01	2,16 ± 0,01	2,17 ± 0,01	2,29 ± 0,01
k_2 ***(x10-4g·mg-1·min-1)***	0,20 ± 0,01	0,20 ± 0,01	0,20 ± 0,01	0,20 ± 0,01	0,23 ± 0,01	0,24 ± 0,01	0,23 ± 0,01	0,22 ± 0,01	0,22 ± 0,01	0,22 ± 0,01	0,22 ± 0,01	0,21 ± 0,01
h (mg·g-1·min-1)	0,68 ± 0,00	0,68 ± 0,00	0,68 ± 0,00	0,71 ± 0,00	1,01 ± 0,00	1,00 ± 0,00	1,00 ± 0,00	1,05 ± 0,00	1,06 ± 0,00	1,04 ± 0,00	1,05 ± 0,00	1,11 ± 0,00
r^2	0,989	0,975	0,987	0,984	0,977	0,971	0,989	0,974	0,961	0,957	0,969	0,959
χ^2												

Metrenco

**Treatment**	**Control**	**AgNPs**	**CuNPs**	**FeNPs**	**Control**	**AgNPs**	**CuNPs**	**FeNPs**	**Control**	**AgNPs**	**CuNPs**	**FeNPs**
q_{exp} ***(mg·g^{-1})***	2,11 ± 0,21	2,17 ± 0,41	2,23 ± 0,19	2,27 ± 0,09	2,17 ± 0,11	2,21 ± 0,26	2,26 ± 0,39	2,29 ± 0,3	2,23 ± 0,21	2,25 ± 0,19	2,29 ± 0,11	2,31 ± 0,13
q_{exp} ***(%)***	52,8 ± 3,1	54,3 ± 3,1	55,5 ± 2,7	57,1± 0,1	54,3 ± 1,2	55,3 ± 2,5	56,5 ± 1,5	57,5± 0,5	55,8 ± 0,3	56,3 ± 1,2	57,3 ± 1,1	58,1 ± 2,5
**Parameters**	**1 Month**				**3 Month**				**6 Month**			
q_e ***(mg·g-1)***	2,27 ± 0,05	2,26 ± 0,05	0,03 ± 0,00	0,03 ± 0,00	2,51 ± 0,08	2,52 ± 0,08	2,56 ± 0,08	2,65 ± 0,08	2,63 ± 0,09	2,69 ± 0,09	2,69 ± 0,09	2,79 ± 0,09
k_2 ***(x10-4g·mg-1·min-1)***	0,03 ± 0,00	0,03 ± 0,00	0,03 ± 0,00	0,03 ± 0,00	0,02 ± 0,00	0,02 ± 0,00	0,02 ± 0,00	0,02 ± 0,00	0,02 ± 0,00	0,02 ± 0,00	0,02 ± 0,00	0,02 ± 0,00
h (mg·g-1·min-1)	0,16 ± 0,00	0,16 ± 0,00	0,16 ± 0,00	0,17 ± 0,00	0,12 ± 0,00	0,12 ± 0,00	0,12 ± 0,00	0,12 ± 0,00	0,12 ± 0,00	0,12 ± 0,00	0,13 ± 0,00	0,13 ± 0,00
r^2	0,983	0,971	0,948	0,972	0,941	0,948	0,962	0,969	0,943	0,967	0,981	0,982
χ^2	0,088	0,121	0,077	0,023	0,015	0,101	0,073	0,099	0,015	0,066	0,179	0,105

Table S2. Kinetic parameters forecasted from the linear evaluation of the intraparticle diffusion kinetic model for the soils studied.

Collipulli

Treatment	Control	AgNPs	CuNPs	FeNPs	Control	AgNPs	CuNPs	FeNPs	Control	AgNPs	CuNPs	FeNPs
Intraparticle diffusion	**1 Month**				**3 Month**				**6 Month**			
q_{e-1} $(mg \cdot g^{-1})$	1,54 ± 0,09	1,59 ± 0,09	1,61 ± 0,13	1,63 ± 0,23	1,56 ± 0,09	1,57 ± 0,12	1,61 ± 0,10	1,64 ± 0,00	1,56 ± 0,11	1,59 ± 0,00	1.62 ± 0,09	1,65 ± 0,01
k_{int-1} $(mg \cdot g^{-1} \cdot min^{1/2})$	0,16 ± 0,02	0,17 ± 0,01	0,18 ± 0,02	0,19 ± 0,03	0,16 ± 0,01	0,18 ± 0,01	0,21 ± 0,03	0,23 ± 0,05	0,17 ± 0,01	0,18 ± 0,01	0,21 ± 0,00	0,23 ± 0,02
C_1 $(mg \cdot g^{-1})$	0,85 ± 0,01	0,83 ± 0,00	0,82 ± 0,00	0,79 ± 0,00	0,83 ± 0,03	0,86 ± 0,02	0,88± 0,09	0,90 ± 0,03	0,83 ± 0,00	0,82 ± 0,00	0,84 ± 0,01	0,86 ± 0,02
r^2	0,978	0,959	0,991	0,992	0,951	0,983	0,934	0,972	0,945	0,939	0,965	0,992

Metrenco

Intraparticle diffusion	**1 Month**				**3 Month**				**6 Month**			
q_{e-1} $(mg \cdot g^{-1})$	1,16 ± 0,09	1,19 ± 0,07	1,21 ± 0,04	1,23 ± 0,32	1,14 ± 0,03	1,20 ± 0,08	1,22 ± 0,03	1,25 ± 0,09	1,16 ± 0,08	1,21 ± 0,02	1,25 ± 0,03	1.28 ± 0,01
k_{int-1} $(mg \cdot g^{-1} \cdot min^{1/2})$	0,23 ± 0,01	0,23 ± 0,00	0,26 ± 0,01	0,28 ± 0,03	0,22 ± 0,01	0,25 ± 0,01	0,27 ± 0,01	0,30 ± 0,03	0,24 ± 0,02	0,25 ± 0,01	0,28 ± 0,01	0,31 ± 0,03
C_1 $(mg \cdot g^{-1})$	0,09 ± 0,00	0,12 ± 0,01	0,14 ± 0,00	0,17 ± 0,01	0,23 ± 0,01	0,24 ± 0,03	0,28 ± 0,01	0,30 ± 0,07	0,24 ± 0,00	0,21 ± 0,04	0,25 ± 0,01	0,32 ± 0,03
r^2	0,955	0,899	0,965	0,942	0,981	0,951	0,956	0,972	0,919	0,995	0,987	0,996
